# Effects of Perfluorotetradecanoic Acid (PFTeDA) and Biostimulants on Soil Bacterial Community Structure and Diversity and the Growth of *Amaranthus cruentus*

**DOI:** 10.3390/ijms27146523

**Published:** 2026-07-22

**Authors:** Małgorzata Baćmaga, Jadwiga Wyszkowska, Edyta Boros-Lajszner, Jan Kucharski, Karolina M. Nowak

**Affiliations:** 1 Department of Soil Science and Microbiology, Faculty of Agriculture and Forestry, University of Warmia and Mazury in Olsztyn, Plac Łódzki 3, 10-727 Olsztyn, Poland; m.bacmaga@uwm.edu.pl (M.B.); edyta.boros@uwm.edu.pl (E.B.-L.); jan.kucharski@uwm.edu.pl (J.K.); 2 Department of Geography, Environmental Geochemistry, Institute for Integrated Natural Sciences, University of Koblenz, Universitätsstraße 1, 56070 Koblenz, Germany; knowak@uni-koblenz.de

**Keywords:** soil, perfluorotetradecanoic acid, biostimulants, soil bacterial community and diversity, 16S rRNA, *Amaranthus cruentus*

## Abstract

Perfluorotetradecanoic acid (PFTeDA), a long-chain per- and polyfluoroalkyl substance (PFAS), is highly persistent and bioaccumulative, yet its effects on soil bacterial communities remain poorly understood. This study evaluated the impact of PFTeDA on the taxonomic composition, diversity, and functional potential of soil bacteria, and assessed whether biostimulants (Shigeki and Aminoprim) could mitigate these effects and improve the growth of *Amaranthus cruentus*. A pot experiment was conducted using Eutric Cambisols soil (pH 4.62), and bacterial communities were analyzed by 16S rRNA gene sequencing. PFTeDA significantly altered bacterial community composition, reducing the abundance of *Actinomycetota*, *Chloroflexota*, and *Gemmatimonadota*, while also changing alpha and beta diversity and predicted functional profiles. Biostimulant application partially alleviated these effects by promoting recovery of the soil bacterial communities and bacteria associated with nitrogen cycling and organic matter degradation, indicating partial restoration of soil ecosystem functions. PFTeDA reduced aboveground biomass but increased root biomass of *A. cruentus*, without affecting leaf greenness (SPAD). Both biostimulants enhanced plant growth and SPAD values, with Aminoprim exerting a stronger effect on shoot biomass and Shigeki stimulating root development. This study demonstrates that PFTeDA affects not only the taxonomic composition of the soil bacterial communities but, above all, its stability and predicted functional potential, representing a key mechanism underlying its impact on the soil–plant system. It also highlights that biostimulation may serve as an effective tool supporting the potential improvement in soils contaminated with persistent organic compounds.

## 1. Introduction

PFASs (per- and polyfluoroalkyl substances) are synthetic compounds characterized by fluorinated carbon chains and polar functional groups (e.g., carboxylate or sulfonate). Due to their exceptional chemical and thermal stability, low surface tension, and resistance to water, grease, and dirt, they are widely used in textiles, food packaging, electronics, coatings, adhesives, aviation fluids, and firefighting foams. Their high environmental persistence, mobility, bioaccumulation potential, and ability to undergo long-range transport have led to their designation as “forever chemicals”. The strong carbon–fluorine bond limits their susceptibility to natural degradation, leading to their global distribution and environmental persistence. PFAS exposure is associated with adverse ecological and human health effects, including oxidative stress and endocrine disruption [1,2,3,4]. Particular concern is associated with long-chain perfluorocarboxylic acids (PFCAs), including PFTeDA (perfluorotetradecanoic acid), due to their potential for bioaccumulation and biomagnification within food webs. Studies show they may cause oxidative stress, tissue damage, and disturbances in physiological and reproductive functions, as well as effects on steroid hormone pathways and gene expression [5]. Soils represent an important environmental sink for PFASs in terrestrial ecosystems. Their presence results from industrial emissions, firefighting foams, fertilizers, composts, irrigation water, and atmospheric deposition. PFASs strongly interact with soil organic matter, which limits mobility but promotes long-term persistence and potential exposure of soil organisms [6]. Global PFAS concentrations vary considerably depending on land use, industrial activity, and local contamination sources. In Europe, ΣPFASs typically range from a few to several tens of ng g^−1^ d.m., exceeding 100 ng g^−1^ in heavily contaminated sites. In Asia, levels range from background values to high industrial concentrations (e.g., 41.82 ng g^−1^ PFOA in eastern China). Elevated values are also reported in Africa (101.39 ng g^−1^) and North America (54.37 ng g^−1^), while Australia/Oceania and South America generally show lower levels, with local hotspots near airports and industrial sites. Even remote regions such as Antarctica shows PFAS contamination, confirming their global environmental distribution [7,8,9]. In Poland, ΣPFAS concentrations generally range from <1–20 ng g^−1^ in agricultural soils to 5–100 ng g^−1^ in urban areas, and may exceed 100 ng g^−1^ in areas affected by industrial activities and waste-related contamination, with locally higher concentrations reported [10].

Bacteria play a crucial role in the functioning of terrestrial ecosystems, for example, in the cycling of elements, decomposition of organic matter, and maintenance of soil fertility. PFTeDA can disrupt the structure and functioning of the soil bacteriobiome and its metabolism. Long-term soil exposure to PFTeDA can disrupt biogeochemical processes and promote the selection of microorganisms possessing antibiotic resistance genes and pathogenic traits [11,12,13,14,15]. At the same time, some bacteria, particularly members of the genera *Pseudomonas* and *Acidimicrobium*, have demonstrated the ability to transform or partially degrade long-chain PFASs, indicating the potential for utilizing biological processes in the remediation of environments contaminated with these compounds. The genomes of these bacteria contain genes encoding enzymes involved in the defluorination of fluorinated compounds, including haloacetate dehalogenase H-1 (dehH1) and haloalkane dehalogenase (dhaA). They also possess multicomponent enzymatic systems including dioxygenases, electron transfer proteins, and 2-halobenzoate 1,2-dioxygenase (CbdA), which may exhibit activity towards fluorinated compounds [16,17].

Despite ongoing research on PFASs, the effects of PFTeDA on soil microbial communities remain poorly understood. Previous research on PFASs has primarily focused on the most widespread and best-characterized members of this group, namely, long-chain perfluorocarboxylic and perfluorosulfonic acid compounds, such as perfluorooctanoic acid (PFOA) and perfluorooctanesulfonic acid (PFOS). These analyses have primarily focused on their environmental persistence, mobility, and impacts on soil and aquatic organisms [18,19,20].

In particular, Senevirathna et al. [19] observed a significant reduction in bacterial diversity in PFOS-contaminated soils and a selective enrichment of specific bacterial taxa, including *Rhodanobacter* and *Chujaibacter*, alongside a decline in the abundance of, among others, RB41 and *Gaiella*. In turn, Cao et al. [3] demonstrated that high PFAS concentrations lead to a substantial reduction in biodiversity and pronounced shifts in bacterial community structure, confirming the key role of these compounds as an important factor shaping the soil microbiome. Understanding the mechanisms underlying the effects of PFTeDAs on soil bacterial communities is of fundamental importance for ecological risk assessment, forecasting changes in ecosystem functioning, and developing effective remediation strategies for soils contaminated with persistent fluorinated pollutants.

Perfluorotetradecanoic acid, a long-chain compound belonging to the group of per- and polyfluoroalkyl substances (PFASs), is characterized by high environmental persistence and the potential to accumulate in soils, which may affect plant growth as well as the structure and activity of soil microbial communities. Previous studies have shown that PFAS compounds can disrupt plant physiological processes and influence the composition and functioning of the soil microbiome; however, the effects of PFTeDA remain poorly understood [21]. Plant biostimulants are widely used in agriculture to stimulate plant growth, improve nutrient use efficiency, and enhance tolerance to environmental stresses. In addition to their direct effects on plant development, they may also influence contaminant transformation and bioavailability in soils by modifying soil properties and microbial activity. Consequently, biostimulants may affect the environmental fate of PFTeDA in soil as well as its potential uptake by plants [22,23].

Accordingly, it was hypothesized that: (i) the presence of PFTeDA in soil may induce change in the structure and diversity of soil bacterial communities as well as affect plant physiological performance; (ii) the application of biostimulants may modulate the responses of soil microorganisms and plants exposed to PFTeDA.

To test these hypotheses, a pot experiment was conducted using *Amaranthus cruentus* as a model plant species. The effects of PFTeDA and selected biostimulants were evaluated based on the analysis of soil bacterial community structure using 16S rRNA gene amplicon sequencing, as well as measurements of plant biomass and leaf greenness index (SPAD index).

## 2. Results

### 2.1. Bacterial Community Composition in Response to Perfluorotetradecanoic Acid and Biostimulants

**Figure 1:**
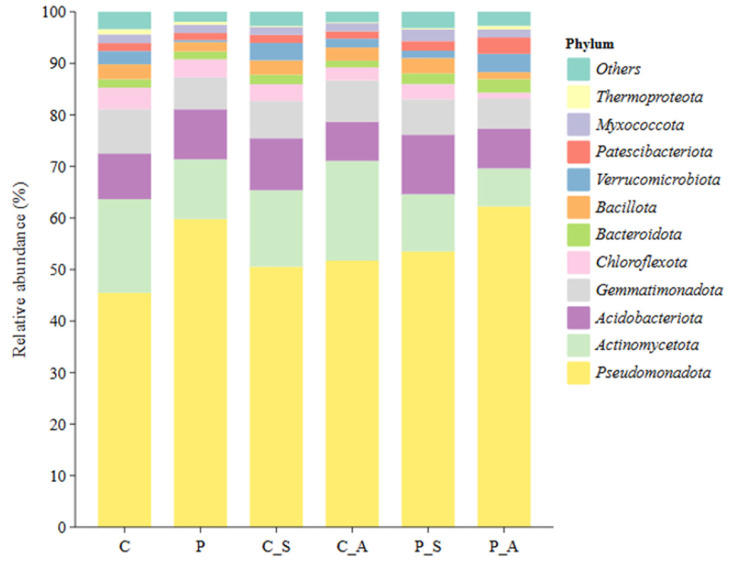
Relative abundance of bacteria (%) at the phylum level in soil contaminated with perfluorotetradecanoic acid (PFTeDA) and supplemented with biostimulants. C—control soil; P—soil treated with perfluorotetradecanoic acid; C_S—soil with the addition of the biostimulant Shigeki; C_A—soil with the addition of the biostimulant Aminoprim; P_S—soil treated with perfluorotetradecanoic acid and with the addition of the biostimulant Shigeki; P_A—soil treated with perfluorotetradecanoic acid and with the addition of the biostimulant Aminoprim.

Analysis of the taxonomic composition of the bacterial community at the phylum level (Figure 1) showed the clear predominance of *Pseudomonadota* in all treatments (45.48–62.26%), followed by the second most abundant phylum *Actinomycetota* (7.36–19.36%). The addition of PFTeDA to soil resulted in an increased relative abundance of *Pseudomonadota* (by 31.46%) and *Acidobacteriota* (by 9.99%) compared with the control, suggesting their potential ability to persist under PFTeDA exposure. Contrastingly, soil treatment with PFTeDA decreased the abundance of *Actinomycetota* (by 36.24%), *Gemmatimonadota* (by 28.18%) and of less abundant phyla such as *Chloroflexota* (by 16.39%), *Bacteroidota* (by 5.51%), *Bacillota* (by 38.26%), *Verrucomicrobiota* (by 84.49%), *Patescibacteriota* (by 11.07%), *Myxococcota* (by 4.17%), and *Thermoproteota* (by 47.03%), indicating their lower relative representation following PFTeDA exposure. The addition of biostimulants also modified bacterial community composition. For instance, in the treatment without PFTeDA, Shigeki enhanced the abundance of *Pseudomonadota* (by 11.15%), Acidobacteriota (by 14.09%), *Bacteroidota* (by 13.48%), *Verrucomicrobiota* (by 31.29%), and *Patescibacteriota* (by 1.98%), while decreasing the relative abundance of other phyla compared to the control. Aminoprim increased the abundance of *Pseudomonadota* (by 13.73%) and *Actinomycetota* (by 6.51%), whereas the remaining phyla showed lower relative abundances. In soil treatments with PFTeDA, biostimulants also induced shifts in bacterial community composition. Shigeki increased the abundance of *Acidobacteriota* by 11.42%, *Bacteroidota* by 32.60%, *Bacillota* by 66.61%, *Verrucomicrobiota* by 254.79%, *Patescibacteriota* by 36.91%, and *Myxococcota* by 40.65%, whilst the abundance of *Pseudomonadota*, *Chloroflexota* and *Thermoproteota* was reduced accordingly by 10.51%, by 15.67%, and by 57.89%. In turn, Aminoprim enhanced the abundance of *Pseudomonadota* (by 4.12%), *Bacteroidota* (by 67.97%), *Verrucomicrobiota* (by 787.68%), *Patescibacteriota* (by 132.68%), and *Thermoproteota* (by 33.60%). Contrastingly, it decreased the abundance of *Actinomycetota* (by 36.50%), *Acidobacteriota* (by 21.05%), *Gemmatimonadota* (by 5.41%), *Chloroflexota* (by 66.16%), *Bacillota* (by 23.67%), and *Myxococcota* (by 4.55%).

**Figure 2:**
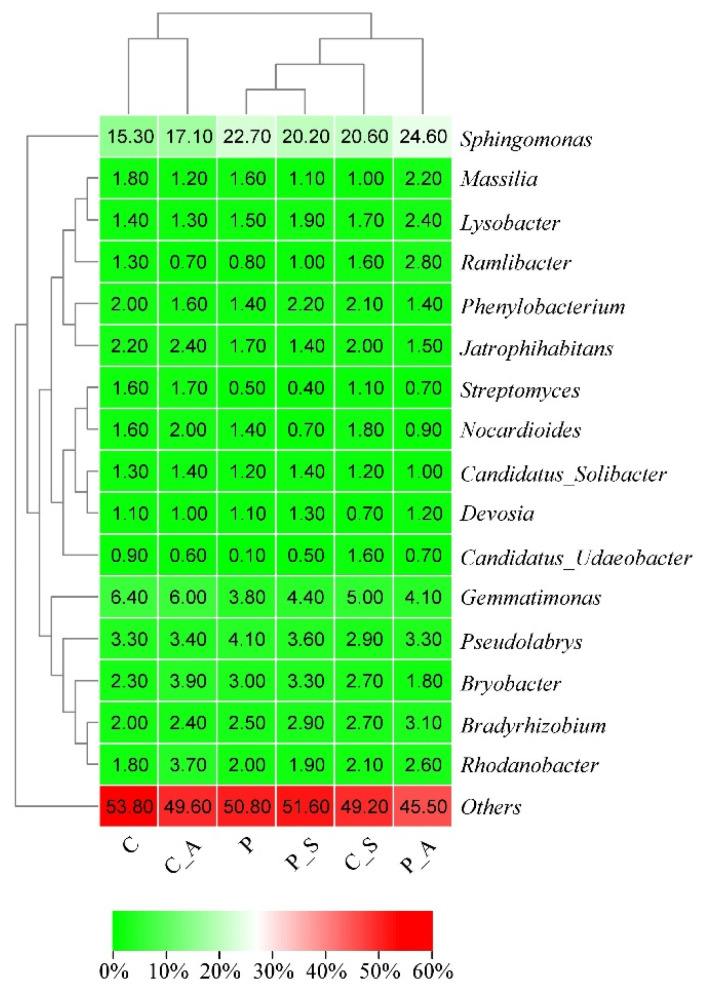
Relative abundance of bacteria at the genus level in soil treated with perfluorotetradecanoic acid (PFTeDA) and supplemented with biostimulants. For abbreviations, see Figure 1.

Analysis of the composition of bacterial communities at the genus level (Figure 2) showed the dominance of the genus *Sphingomonas* (15.22–24.43%) in all treatments. Other genera like *Gemmatimonas* (3.81–6.35%), *Pseudolabrys* (2.89–4.05%), and *Bradyrhizobium* (1.96–3.09%) were also relatively abundant within the bacterial community. Compared with the control soil (C), without the addition of perfluorotetradecanoic acid or biostimulants, PFTeDA application (P-only) increased the relative abundance of the bacterial genera *Sphingomonas* (32.51%), *Bradyrhizobium* (21.19%), *Pseudolabrys* (18.02%), *Lysobacter* (8.11%), *Bryobacter* (22.18%), and *Rhodanobacter* (7.6%) At the same time, a decrease in the abundance of *Ramlibacter* by 69.74%, *Gemmatimonas* by 66.67%, *Phenylobacterium* by 41.13%, *Jatrophihabitans* by 31.10%, *Nocardioides* by 20.15%, *Candidatus_Solibacter* by 10.26%, and *Massilia* by 10.76% was observed. In uncontaminated soil, Shigeki increased the abundance of *Sphingomonas* (by 19.48%), *Bradyrhizobium* (by 19.55%), *Ramlibacter* (by 14.01%), and *Lysobacter* (by 10.69%), while decreasing the abundance of other genera compared with the control soil (C). Aminoprim increased the relative abundance of *Bryobacter*, *Nocardioides*, *Bradyrhizobium*, and *Rhodanobacter* by 3.92–42.48%, while reducing the relative abundance of other genera. In PFTeDA-treated soil, the effect of biostimulants on bacterial genus composition differed from that observed in untreated soils. ent. Shigeki increased the abundance of *Gemmatimonas* (by 13.60%), *Bradyrhizobium* (by 14.44%), *Ramlibacter* (by 23.55%), *Phenylobacterium* (by 35.53%), and *Candidatus_Udaeobacter* (by 72.15%), while decreasing the relative abundance of *Nocardioides* (by 90.41%). Aminoprim promoted the growth of *Sphingomonas* (by 14.45%), *Massilia* (by 34.05%), *Gemmatimonas* (by 12.97%), *Streptomyces* (by 37.55%), *Bradyrhizobium* (by 25%), *Ramlibacter* (by 75%), and *Candidatus_Udaeobacter* (by 81%), whereas it reduced the relative abundance of *Bryobacter* (by 49.67%), *Nocardioides* (by 40.40%), *Candidatus_Solibacter* (by 14.78%), and *Pseudolabrys* (by 13.46%).

**Figure 3:**
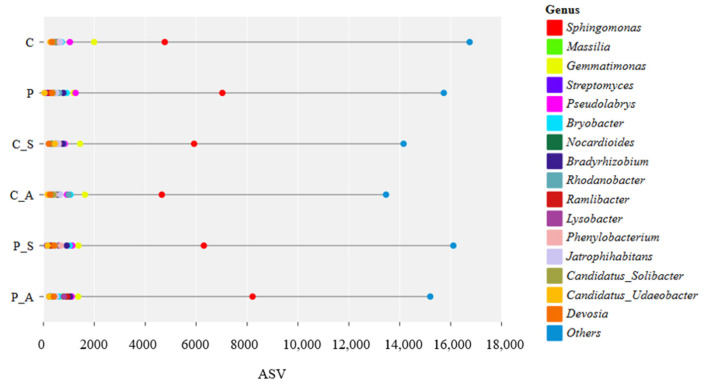
Abundance of amplicon sequence variants (ASVs) representing the dominant bacterial genera in soil treated with perfluorotetradecanoic acid (PFTeDA) and supplemented with biostimulants. For abbreviations, see Figure 1.

Among the identified taxa, the genus *Sphingomonas* exhibited the highest ASV count across all treatments (Figure 3), and its highest abundance was noticed in soil treated with both PFTeDA and Aminoprim (P_A: 8217). *Sphingomonas* was also abundant in descending order for PFTeDA treatment (P: 7030), PFTeDA + Shigeki treatment(P_S: 6303), Shigeki treatment (C_S: 5920), control soil (C: 4767), and the least abundant in the Aminoprim treatment (C_A: 4651). The remaining bacterial genera were considerably less represented, with ASV counts below 2000. Relatively higher abundances were found for the following genera *Massilia*, *Gemmatimonas*, *Streptomyces*, and *Pseudolabrys*; however, their abundance was clearly lower than that of *Sphingomonas*. Overall, a similar distribution pattern of less abundant genera was observed across treatments, with no pronounced shifts in their relative abundance. To sum it up, the bacterial community composition was shaped primarily by the genus *Sphingomonas*, suggesting its highest adaptability to all tested conditions compared to other genera.

**Figure 4:**
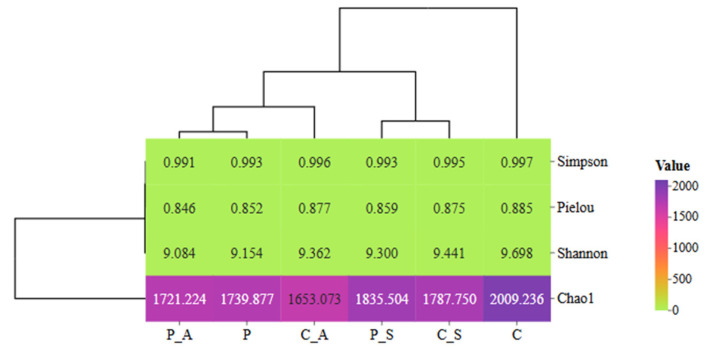
Alpha diversity of bacteria in soil treated with perfluorotetradecanoic acid (PFTeDA) and supplemented with biostimulants. For abbreviations, see Figure 1.

Analysis of alpha-diversity indices showed that treatment with PFTeDA exposure resulted in a reduction in bacterial community richness, diversity, and evenness (Figure 4). In PFTeDA-treated soil (P), the Chao1 index value was 1739.877 and was lower compared to the control soil (2009.236), indicating a decrease in bacterial taxonomic richness. The less pronounced decrease in the Shannon index value (from 9.698 to 9.154) and the Pielou index value (from 0.885 to 0.852) was also recorded under PFTeDA exposure conditions, indicating a reduction in both the diversity and evenness of bacterial community composition. The Simpson index, however, was relatively high and showed only minor variation among treatments (0.991–0.997). The greatest changes in alpha-diversity indices were observed in P_A treatment, in which the Chao1 (1721.224), Shannon (9.084), and Pielou (0.846) index values were the lowest among all PFTeDA treatments. The P_S treatment exhibited higher Chao1 (1835.504) and Shannon (9.300) index values compared with the PFTeDA-only treatment (P). The application of biostimulants also affected alpha-diversity patterns in control soils (without PFTeDA exposure). Compared with the control treatment without biostimulant, both C_S and C_A treatments showed decreased values of alpha-diversity indices, particularly the Chao1 index. In PFTeDA-treated soils, biostimulants also influenced bacterial diversity patterns. In the P_S treatment, higher Chao1, Shannon, and Pielou index values were recorded compared to the P treatment. In contrast, the P_A treatment showed the lowest values of these indices among all PFTeDA-exposed treatments.

**Figure 5:**
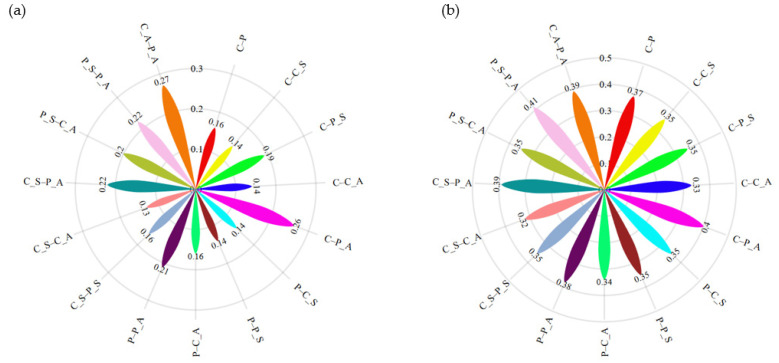
Beta diversity of bacteria in soil treated with perfluorotetradecanoic acid (PFTeDA) and supplemented with biostimulants: (**a**) Bray–Curtis dissimilarity; (**b**) Jaccard distance. Distance differences between the analyzed soil samples: C–P, C–C_S, C–P_S, C–C_A, C–P_A, P–C_S, P–P_S, P–C_A, P–P_A, C_S–P_S, C_S–C_A, P_S–P_A, C_A–P_A. C—control soil; P—soil treated with perfluorotetradecanoic acid; C_S—soil with the addition of the biostimulant Shigeki; C_A—soil with the addition of the biostimulant Aminoprim; P_S—soil treated with perfluorotetradecanoic acid and with the addition of the biostimulant Shigeki; P_A—soil treated with perfluorotetradecanoic acid and with the addition of the biostimulant Aminoprim.

Soil treatment with PFTeDA and the application of biostimulants significantly modified the beta diversity of bacterial communities (Figure 5a,b). The Bray–Curtis dissimilarity index ranged from 0.129 to 0.27, indicating low to moderate differentiation among the analyzed bacterial communities (Figure 5a). The highest dissimilarity was recorded between the C_A and P_A soil samples (0.267), and the smallest differences between C_S and C_A (0.129). Comparisons among the remaining treatments revealed moderate differences in bacterial community composition. The Jaccard dissimilarity coefficient ranged from 0.323 to 0.412, indicating moderate differentiation between the analyzed treatments (Figure 5b). The highest value was found between the P_S and P_A samples (0.412), and the lowest between C_S and C_A (0.323). The remaining treatment comparisons also showed moderate differences in bacterial community composition.

**Figure 6:**
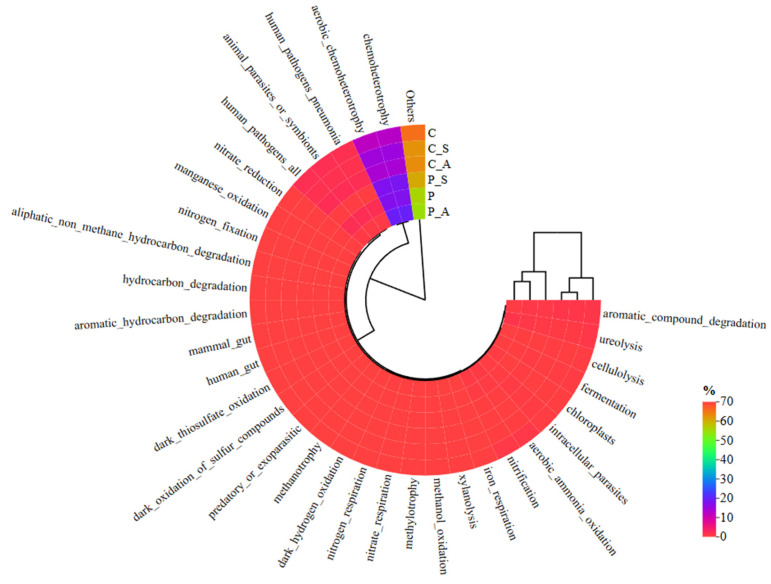
Predicted metabolic functions of bacteria in soil treated with perfluorotetradecanoic acid (PFTeDA) and supplemented with biostimulants. For abbreviations, see Figure 1.

The analysis of the predicted functional potential of the bacterial microbiome revealed a predominance of metabolic functions predicted from taxonomic profiles, including chemoheterotrophy (12.47–19.55%) and aerobic chemoheterotrophy (11.98–19.34%) (Figure 6). In PFTeDA-treated soils, relative representation of these predicted functional groups was observed compared to the control sample, suggesting an increased predicted potential for the utilization of organic compounds as energy and carbon sources by bacterial communities. The other groups with specialized metabolic capabilities were characterized by lower abundance. Bacteria associated with the transformation of aromatic compounds accounted for 0.5–1.1%, while those involved in nitrogen compound transformations (including nitrate reduction, nitrification, and ammonia oxidation) accounted for 0.11–0.70%. Fermentative bacteria amounted to 0.15–0.42%, ureolytic bacteria to 0.556–1.25%, and polysaccharide-degrading bacteria to 0.11–0.30%. Taxa with predicted methylotrophic, methanol-oxidizing, hydrocarbon-degrading, and manganese-oxidizing functions occurred at very low relative abundances (<0.1%). Overall, the results indicate the dominance of predicted aerobic heterotrophic metabolism and a lower contribution of less represented functional groups associated with nitrogen cycling and the degradation of more complex organic compounds.

### 2.2. Response of Amaranthus cruentus to Perfluorotetradecanoic Acid and Biostimulants

**Figure 7:**
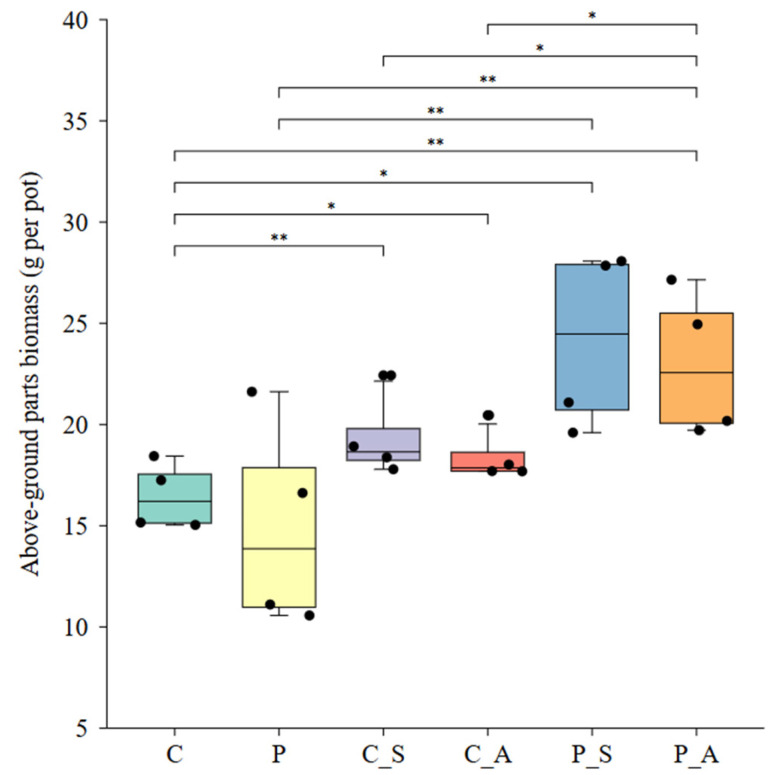
Impact of soil treatment with perfluorotetradecanoic acid (PFTeDA) and biostimulant addition on the aboveground biomass of *Amaranthus cruentus*. *—statistically significant at *p* < 0.05; **—statistically significant at *p* < 0.01. For abbreviations, see Figure 1.

**Figure 8:**
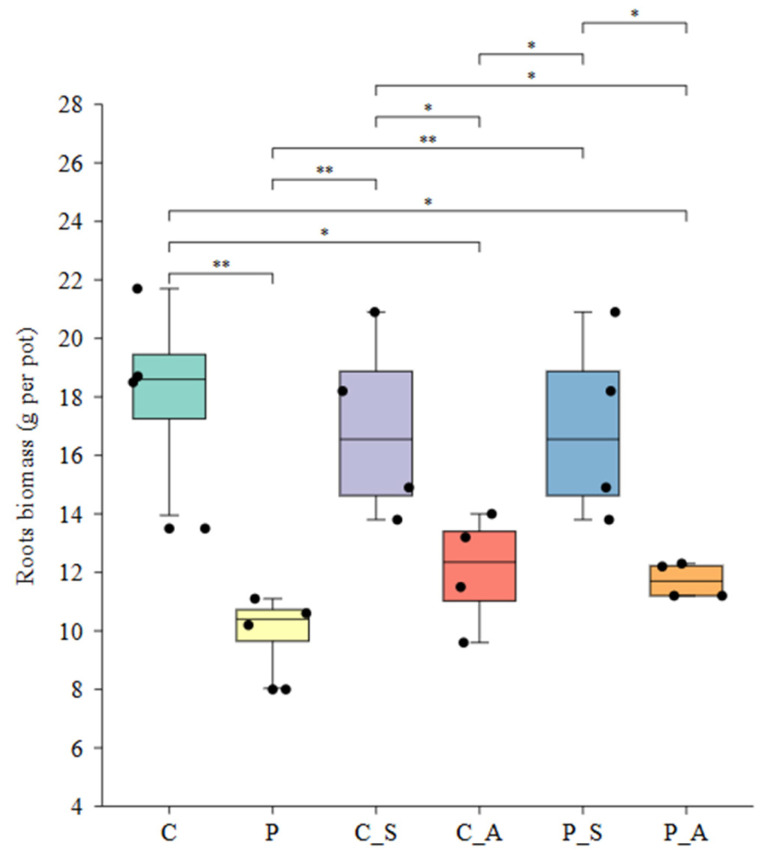
Impact of soil treatment with perfluorotetradecanoic acid (PFTeDA) and biostimulant addition on the root biomass of *Amaranthus cruentus*. *—statistically significant at *p* < 0.05; **—statistically significant at *p* < 0.01. For abbreviations, see Figure 1.

**Figure 9:**
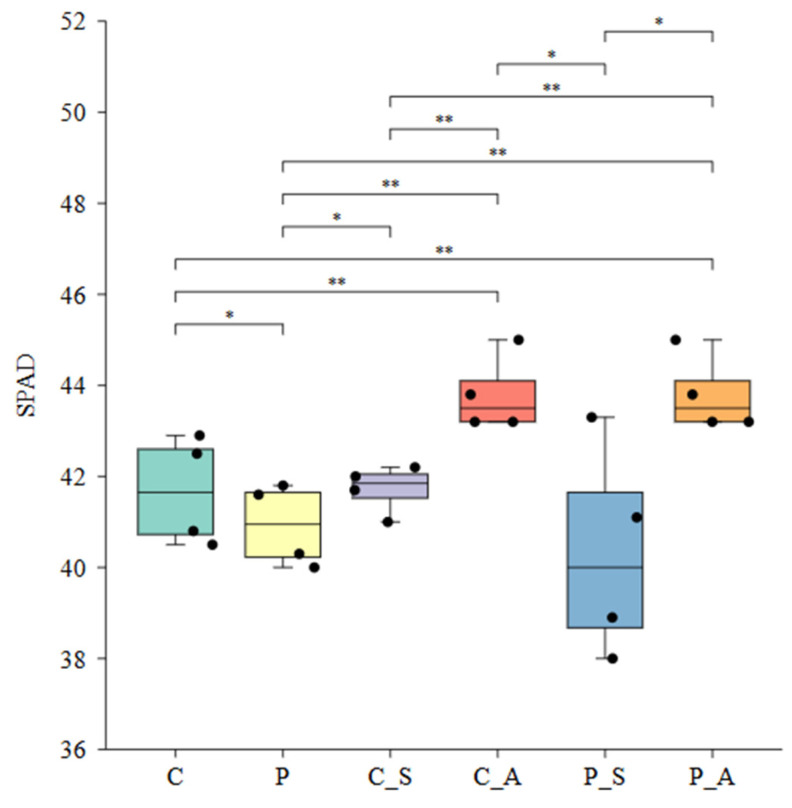
Impact of soil treatment with perfluorotetradecanoic acid (PFTeDA) and biostimulant addition on the leaf greenness index (SPAD) of *Amaranthus cruentus*. *—statistically significant at *p* < 0.05; **—statistically significant at *p* < 0.01. For abbreviations, see Figure 1.

Analysis of the impact of PFTeDA and the biostimulants Shigeki and Aminoprim on the growth of *Amaranthus cruentus* revealed varied effects depending on the soil treatment applied (Figure 7, Figure 8 and Figure 9).

Soil treatment with PFTeDA affected the aboveground biomass of plants (Figure 7). In the treatment without biostimulant addition, a lower biomass in control soil (C; 16.473 g pot^−1^) was higher than that observed in PFTeDA-treated soil (14.980 g pot^−1^), suggesting a potential inhibitory effect of PFTeDA exposure on plant growth. The application of the biostimulant Shigeki increased aboveground biomass in C_S and P_S treatments, respectively, by 17.66% and 23.28%. A much stronger stimulatory effect on above-ground biomass was observed for Aminoprim treatments; i.e., about a 24.63% increase in both C_A and P treatments. These results indicate that Aminoprim was particularly effective in promoting plant aboveground biomass under the tested conditions.

Soil treatment with only PFTeDA (P-treatment) reduced one plant root biomass by 21.51% compared with the control soil (C-treatment), suggesting potential adverse effects of PFTeDA exposure on root development (Figure 8). Contrastingly, application of the biostimulant Shigeki significantly increased root biomass in the C_S-treatment by 30.43% compared with the control (C-treatment). However, in the P_S treatment, this effect was considerably weaker, with only a 5.73% increase observed compared with the P-treatment. A similar observation was made for the biostimulant Aminoprim—in C_A treatment, root biomass increased by 29.56%, while in P_A treatment a 2.52% increase was recorded compared to the P treatment. These results indicate that both biostimulants promoted root biomass production under control conditions, while PFTeDA exposure reduced their effectiveness in stimulating root development.

The leaf greenness index (SPAD), used as an indirect indicator of leaf chlorophyll content, showed a limited response to PFTeDA exposure and biostimulant application (Figure 9). In the P-treatment, a 1.8% decrease in SPAD values compared with C-treatment was observed. No significant changes in the SPAD value were noticed after addition of the biostimulant Shigeki to soil in both C_S- and P-treatments. A more pronounced effect was observed in soils treated with the biostimulant Aminoprim treatments.

## 3. Discussion

### 3.1. Bacterial Community Composition in Response to Perfluorotetradecanoic Acid and Biostimulants

The obtained results indicate that PFTeDA is a factor influencing bacterial community composition due to its high persistence, bioaccumulative potential, and potential toxicity to living organisms [4]. The increased prevalence of the *Pseudomonadota* phylum exposed may be associated with the selective enrichment of taxa capable of persisting under contaminant stress. This phylum has been documented to grow more vigorously than other phyla in the presence of PFOA in soils and of antibiotics in a wastewater plant [13]. Contrastingly, reduced abundances of *Actinomycetota*, *Chloroflexota* and *Gemmatimonadota* phyla under the influence of PFTeDA showed their low tolerance. This is likely attributable to the high persistence and bioaccumulative nature of PFTeDA, which can disrupt cell membrane integrity, inhibit enzymatic activity, and induce oxidative stress. Since long-chain PFASs are highly resistant to biodegradation, exposure to PFTeDA may contribute to shifts in bacterial community structure and reduced representation of sensitive taxa [3,12]. These phyla are known to contribute to soil organic matter transformation and nutrient cycling; therefore, changes in their relative abundance may influence soil ecological processes and potentially affect soil quality [24]. Taxa that are sensitive to persistent and hydrophobic contaminants, such as PFTeDA, may exhibit reduced competitiveness under contamination pressure, resulting in changes in ecological niches within the bacterial community [25], and as a consequence, ecological niches within the bacteriobiome are downsized. The changes in the bacteriobiome can cause ecological imbalance and alter soil ecosystem functions. The limitation of the development of the remaining bacterial phyla additionally suggests disruption of the slower organic matter cycling processes and the sensitivity of these taxa to chemical pressure [26,27]. Notably, the negative effects associated with PFTeDA exposure appeared to be less pronounced following the application of the Shigeki biostimulant [28]. The soil organic matter in nature is negatively charged (-COOH) [6]; the addition of polyvalent cations (positively charged) can change the negative charge of soil organic matter towards positive and thus foster the sorption of negatively charged PFTeDA (-COOH). Consequently, a lower fraction of PFTeDA may remain available in the soil solution, potentially reducing its bioavailable to living soil organisms.

The predominance of the *Sphingomonas* genus in all treatments and in particular after PFTeDA application suggests its high tolerance to all tested conditions. This genus has been proven to tolerate diverse xenobiotics, to degrade aromatic compounds, and to possess a rigid glycosphingolipid-based cell membrane [29]. Its increase indicates the selection of tolerant microorganisms, potentially involved in co-metabolic transformations of contaminants and the stabilization of the bacteriobiome [30]. The genus *Sphingomonas* is widely distributed in natural soils and constitutes a frequently detected bacterial group in terrestrial ecosystems. Its ecological persistence is associated with high metabolic versatility, the capacity to utilize a wide range of organic substrates, and efficient adaptive mechanisms that facilitate survival under environmental stress. Members of this genus are frequently enriched in chemically disturbed soils, indicating their potential contribution to microbial community adaptation under changing environmental conditions. Thus, the increased relative abundance of *Sphingomonas* following PFTeDA exposure and biostimulant application may indicate enhanced tolerance to chemical stress and a selective advantage of these bacteria in disturbed soil environments [31,32,33]. Simultaneously, the increased abundances of *Bradyrhizobium*, *Pseudolabrys*, and *Devosia* may indicate the preservation of functions associated with nitrogen cycling and plant–microorganism interactions. However, functional analysis indicates disturbances in the nitrogen cycle, including nitrification, ammonia oxidation, and nitrate reduction, as well as reduction in the enzymatic potential associated with nitrogen and phosphorus cycles [13].

The diversity of the bacterial community composition has changed under the influence of PFTeDA as shown in alpha- and beta-diversity indices. The decrease in Chao1, Shannon, and Pielou index values indicates a reduction in taxonomic richness and an increase in the dominance of selected groups, which is typical of strong chemical stressors leading to environmental selection and the elimination of sensitive taxa [34,35]. Beta-diversity analysis also confirmed significant changes in the composition of bacterial communities, with higher Jaccard distance values relative to Bray–Curtis indicating that PFTeDA primarily causes the replacement of taxa, rather than merely changes in their abundance. This signifies a strongly selective effect, resulting in the elimination of rare and sensitive groups of microorganisms, which is characteristic for persistent organic pollutants [36,37].

Analysis of the predicted metabolic potential of the bacteriome indicated the predominance of chemoheterotrophic functions in both control and PFTeDA-treated soils, with an increased predicted contribution of these functions under PFTeDA exposure. These findings may suggest a shift in the predicted functional profile of microbial communities towards enhanced utilization of readily available organic compounds and potential alterations in carbon cycling processes. At the same time, the lower predicted representation of functions associated with nitrogen cycling, including nitrification, denitrification, and nitrogen fixation, may indicate potential disturbances in key soil biogeochemical processes. Such changes could contribute to alterations in soil fertility and ecosystem functioning, while the observed enrichment of microorganisms with predicted capacities for degrading or tolerating toxic compounds may reflect partial microbial adaptation to contamination stress [34,35,38].

The applied biostimulants also modified the composition and diversity of the bacteriobiome, with the scale and direction of changes depending on biostimulant type and the presence or absence of PFTeDA. The results confirm that biostimulants can influence soil microbial community structure, potentially affecting taxon interactions, niche availability, and predicted functional potential [39,40,41]. In soil without PFTeDA addition, both biostimulants increased the abundance of the *Pseudomonadota* phylum, which is a copiotrophic microorganism characterized by rapid responses to nutrient availability and environmental changes [36,42,43]. Shigeki also increased the relative abundance of *Acidobacteriota*, *Bacteroidota*, *Verrucomicrobiota*, and *Patescibacteriota*; this suggests shifts in bacterial community structure that may be associated with change in organic matter transformation and nutrient cyclin potential [44,45,46]. Aminoprim exhibited a more selective effect, increasing *Actinomycetota*, a phylum associated with the decomposition of complex organic compounds and soil organic matter transformation [13,47,48,49]. In PFTeDA-treated soil, both biostimulants possibly mitigated the negative effects of the stressor, but through different ecological mechanisms [4]. Shigeki promoted the reconstruction of a more balanced bacterial microbiome structure, increasing the abundance of the phyla *Bacteroidota*, *Bacillota*, *Verrucomicrobiota*, *Patescibacteriota*, and *Myxococcota* while it inhibited the dominant phylum *Pseudomonadota*, indicating partial reconstruction of complex trophic interactions [50,51,52]. On the other hand, Aminoprim promoted taxa that respond rapidly to improved environmental conditions, at the expense of groups maintaining organic matter cycling. The rapidly growing taxa, however, may limit the full recovery of the bacteriobiome composition [36,37,39].

At the genus level, both biostimulants increased the relative abundance of ecologically relevant bacterial genera, including *Sphingomonas*, *Bradyrhizobium*, *Ramlibacter*, *Lysobacter*, and *Devosia*. The increase in the abundance of *Sphingomonas* may indicate the selective enrichment of taxa capable of persisting under PFTeDA exposure rather than the direct development of tolerance. After the growth inhibition of PFTeDA-sensitive genera, the competition for nutrient resources is lower; therefore, more nutrients will be available for the growth of stronger genera. *Bradyrhizobium* and *Devosia* may enhance nitrogen cycling and plant–bacteria interactions, while *Lysobacter* may limit the development of phytopathogens through the production of antimicrobial metabolites [33]. The biostimulants also exhibited a stabilizing effect, limiting the disturbances of the bacteriobiome structure induced by PFTeDA and supporting its recovery. The obtained results highlight that soil ecosystem resilience depends on the maintenance of a complex bacterial community structure and its predicted functional balance, rather than merely on the presence of individual bacterial groups [51,52,53,54,55,56].

Alpha- and beta-diversity analysis indicates that the impact of biostimulants did not always lead to an increase in overall microbiome diversity. In some treatments, a decrease in community evenness was observed, which resulted from the selective growth of chosen bacterial groups. This means that the assessment of biostimulation effectiveness should consider not only changes in microorganism abundance but also the stability and ecological balance of the entire community [57,58,59,60].

The biostimulants exhibited varied potential for modulating the bacteriobiome both under natural conditions and under PFTeDA pressure. Shigeki acted more regeneratively, supporting the restoration of a balanced taxonomic structure and increasing functional diversity, while Aminoprim more strongly stimulated opportunistic and fast-growing bacteria. These results indicate that the choice of biostimulant should be adapted to the application goal, encompassing soil reclamation, improvement of its biological fertility, or activation of microbiological processes.

### 3.2. Response of Amaranthus cruentus to Perfluorotetradecanoic Acid and Biostimulants

This study demonstrated that PFTeDA exerted varied effects on the growth of *A. cruentus*, causing only a reduction in the aboveground biomass of plants. This may indicate disturbances in physiological processes, such as oxidative stress, limited mineral nutrient uptake, and disrupted water management [61,62]. Due to its high persistence and sorption to organic matter, PFTeDA can persist over the long term, accumulating mainly in plant organs and modifying their metabolism [63,64]. An increased root biomass can be the result of an adaptive response of *A. cruentus* to environmental stress, involving the intensification of the root system to compensate for limited water and nutrient uptake [61]. The SPAD value did not undergo significant changes, indicating the absence of marked disturbances in chlorophyll synthesis and the functioning of the photosynthetic apparatus. As reported by Karamat et al. [64], photosynthetic responses to PFASs are strongly dependent on species, dose, and exposure time; therefore, these changes may not constitute an early indicator of toxicological stress. The reduction in shoot biomass and the increase in root biomass observed in response to PFTeDA exposure may reflect a complex physiological adjustment of plants to chemical stress. One possible explanation is the preferential allocation of assimilates to the root system, which may support water and mineral nutrient acquisition under adverse conditions. However, increased root biomass may also be associated with the direct effects of PFTeDA on plant growth regulation, including disturbances in hormonal pathways involving auxin, abscisic acid, and cytokinins, which control the balance between root and shoot development [65]. Furthermore, long-chain PFASs, including PFTeDA, have a tendency to accumulate in roots and exhibit limited translocation to aboveground tissues, potentially contributing to root-localized stress responses and disturbances in mineral nutrient homeostasis [66,67]. Thus, the observed increase in root biomass may represent an adaptive response, but it could also reflect PFTeDA-induced hormonal imbalances and disruptions of physiological homeostasis.

An important goal of this research was to assess the impact of the biostimulants Shigeki and Aminoprim on the growth of *Amaranthus cruentus* after the addition of PFTeDA to soil. Both biostimulants increased aboveground and root biomass in both the control and PFTeDA-treated soil. Aminoprim stimulated the growth of aboveground plant parts whilst Shigeki stimulated the development of the root system. The biostimulants containing amino acids, seaweed extracts, and humic substances were reported to improve nutrient uptake by plants, intensify photosynthesis, and regulate plant hormonal balance [68]. Additionally, they increase tolerance to abiotic stress by activating the antioxidant system, stabilizing cell membranes, and reducing lipid peroxidation and malondialdehyde accumulation [69,70]. This includes, among others, an increase in the activity of enzymes such as superoxide dismutase, catalase, and peroxidases, which reduce oxidative cell damage [71]. The observed increase in root biomass after the application of Shigeki may result from the stimulation of meristem activity and the development of lateral roots, which improves water and mineral nutrient uptake [72]. Consecutively, the slight increase in the SPAD value after the application of Aminoprim indicates improved nitrogen supply and intensified chlorophyll biosynthesis, which promotes photosynthetic efficiency and biomass accumulation [73].

In summary, PFTeDA exhibited only a minor effect on the growth of *A. cruentus*, manifested primarily by changes in aboveground plant biomass. The application of the biostimulants Shigeki and Aminoprim contributed to the improvement of plant growth and development parameters and supported their physiological condition. The obtained results indicate that the use of biostimulants may constitute a promising strategy for supporting crop production on soils contaminated with persistent perfluoroalkyl compounds [64].

## 4. Materials and Methods

### 4.1. Experimental Design

**Figure 10:**
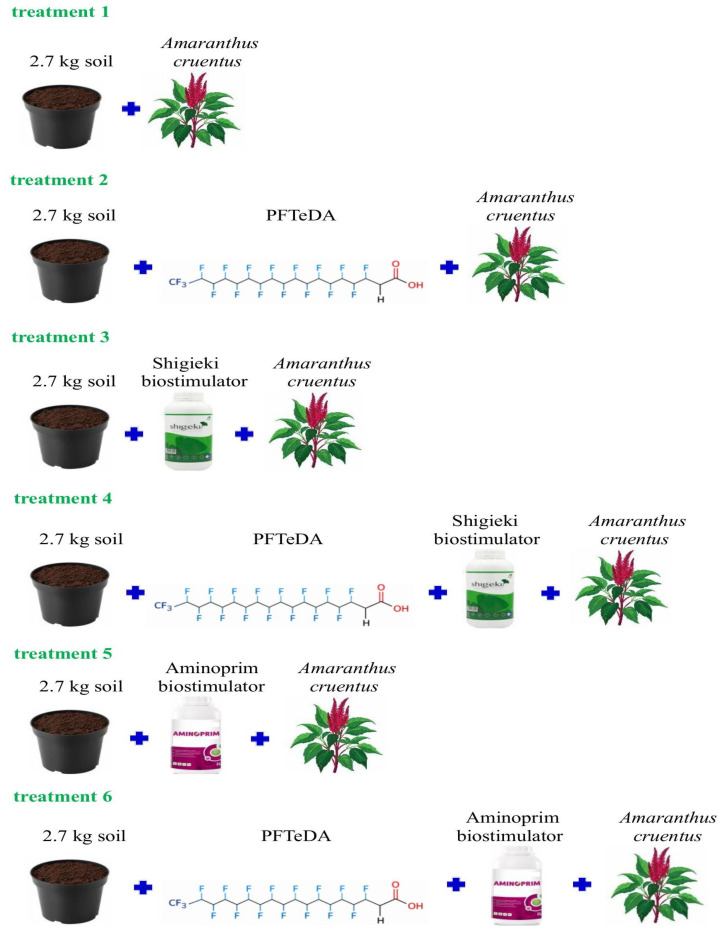
Combinations used in the experiment.

The pot experiment was conducted in a vegetation hall (4 replicates) located at the University of Warmia and Mazury in Olsztyn. Six different treatments were prepared as shown in Figure 10. The reference was soil without the addition of perfluorotetradecanoic acid and biostimulants. The model plant was *A. cruentus*. *A. cruentus* was selected due to its high adaptability to diverse environmental conditions, including exposure to chemical stressors. This species exhibits considerable tolerance to contaminant-induced environmental stress and has the capacity to accumulate pollutants within its tissues. These characteristics indicate its potential suitability for studies to phytoremediation and the assessment of plant responses in contaminated soils [74,75]. Plastic pots with a capacity of 3.0 dm^3^ were filled with 2.7 kg of reference soil (dry matter, d.m.). In the PFTeDA treatments, PFTeDA was applied once as an aqueous solution at a rate of 160 ng kg^−1^ d.m. soil. The concentration of perfluorotetradecanoic acid applied in this study was selected based on previously reported environmental levels [76,77,78,79,80], which range from 1.0 to 100 ng kg^−1^ dry soil mass. Given the high persistence and accumulation potential of long-chain PFASs in soils, even low concentrations of these compounds may induce measurable alterations in biological and physicochemical processes within the soil environment. The biostimulants Shigeki (25 dm^3^ ha^−1^) and Aminoprim (3.5 dm^3^ ha^−1^) were applied twice: on the day the experiment was established and on day 25. The control sample consisted of soil without the addition of the chemical compound and biostimulants. On day 0 of the experiment, the following mineral fertilizers were added to the soil (mg kg^−11^ dry matter, d.m.): N—70 mg (as CO(NH_2_)_2_), P—30 mg (as KH_2_PO_4_), and K—100 mg (as KH_2_PO_4_ and KCl). Thereafter, the soil was sown with plants, and then soil moisture was adjusted to 50% of capillary water capacity. After the germination of A. cruentus (BBCH 9—Biologische Bundesanstalt, Bundessortenamt und Chemische Industrie), thinning was performed to obtain a comparable amount of biomass between all pots, and only 4 plants were kept in each pot. At the BBCH 29 growth stage of A. cruentus, the leaf greenness index (SPAD) was determined using a KONICA MINOLTA, Inc. chlorophyll meter (Chiyoda, Japan). On the 50th day of the experiment, when A. cruentus was at the BBCH 39 growth stage, the plants were harvested, and the dry mass of aboveground parts and roots was determined. On the same day, soil was sampled for phylogenetic analyses (16S rRNA) using next-generation sequencing (NGS). The experiment was conducted in a vegetation hall from May to July 2025. During the experimental period, the average temperature was 16.5°C, with an average photoperiod of 16 h. At the end of the experiment, the entire soil volume from each pot was transferred into sterile containers, and residual root fragments were carefully removed. The soil was thoroughly homogenized to obtain representative material for each sample. Following sample preparation, the soil samples were immediately sealed and stored at −32°C until further analyses. Throughout the experiment, pots were regularly repositioned within the vegetation hall to minimize potential positional effects.

### 4.2. Reference Soil and Its Characteristics

**Figure 11:**
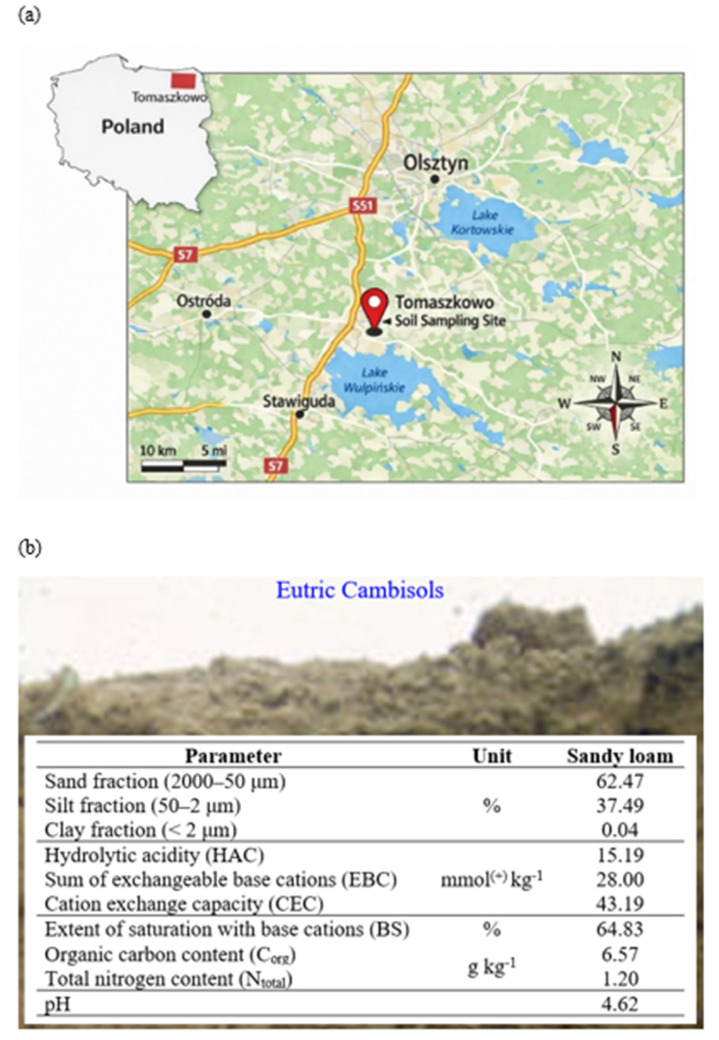
Location of the soil sampling site (**a**) and soil physicochemical properties (**b**).

The reference soil, classified as Eutric Cambisols according to the WBR system (World Reference Base for Soil Resources) [81], was collected from topsoil arable land (0–20 cm) in the village Tomaszkowo (53° 43′ N, 20° 24′ E) located in the administrative district of Stawiguda Commune, Olsztyn County (see Figure 11a). The soil was then transported to the Didactic and Experimental Hall of the University of Warmia and Mazury in Olsztyn (53° 71′ N, 20° 43′ E), where it was sieved at 5 mm to obtain a homogeneous plant- and stone-free fraction. The physicochemical properties of the soil were determined in the lab according to the methods as described previously by Wyszkowska et al. [82,83], and its parameters are presented in Figure 11b.

### 4.3. Characteristics of Perfluorotetradecanoic Acid and Biostimulants

The PFTeDA (PESTANAL^®^ analytical standard, CAS number: 376-06-7, EC:206-803-4, purity ≥96.0%) was purchased from Sigma-Aldrich Production GmbH, Buchs, Switzerland. The physicochemical properties of PFTeDA are presented in Table 1.

Two biostimulants, Shigeki and Aminoprim, were applied to provide nutrients and amino acids needed for the growth of *A. cruentus*. Shigeki was manufactured by Futureco Bioscience S.A. (Barcelona, Spain). It is a plant biostimulant used under stress conditions. It contributes to better root system development and increases plant resistance to stress caused by high temperatures, hail, and stress related to protective treatments. It contains concentrated extract of the seaweed *Ascophyllum nodosum* (10%), macronutrients: P—10% (P_2_O_5_), K—10% (K_2_O), and micronutrients: B—0.13%, Mo—0.05%, Cu—0.10% (EDTA-Cu), Fe—0.20% (EDTA-Fe), Mn—0.20% (EDTA-Mn), and Cu—0.20% (EDTA-Cu). Aminoprim is an organic biostimulant manufactured by Intermag (Olkusz, Poland). It contains a highly concentrated dose of biologically active amino acids and short-chain peptides of natural origin. It includes the following amino acids: glycine—11.6%, proline—6.5%, alanine—5.8%, glutamic acid—5.5%, hydroxyproline—4.5%, ornithine—2.6%, lysine—2.2%, aspartic acid—2.0%, leucine—2.0%, hydroxylysine—1.5%, phenylalanine—1.2%, isoleucine—0.6%, methionine—0.6%, tyrosine—0.6%, arginine—0.5%, histidine—0.4%, serine—0.2%, and threonine—0.1%.

### 4.4. DNA and Bioinformatics Analysis

Isolation and identification of genomic DNA, as well as bioinformatics analysis, were performed by Novogene (UK) Company Limited (Cambridge, UK). Bacterial community composition identification was based on 16S rRNA gene analysis using specific primers listed in Table 2.

DNA was extracted from 0.5 g of homogenized soil collected from composite samples representing each experimental treatment. PCR reactions were performed in a volume of 15 μL using Phusion High-Fidelity PCR Master Mix (Thermo Fisher Scientific, Waltham, MA, USA), 0.2 μM of each primer, and approximately 10 ng of DNA. The program included initial denaturation (98 °C, 1 min), 30 cycles of denaturation (98 °C, 10 s), annealing (50 °C, 30 s), and elongation (72 °C, 30 s), followed by final elongation (72 °C, 5 min). PCR products were purified, sequencing libraries were prepared, their quality and quantity were assessed (Qubit, qPCR, bioanalyzer) and finally sequenced on the NovaSeq 6000 platform (Illumina, San Diego, CA, USA). Reads were assigned to samples based on barcodes, primer sequences were removed, and reads were merged using FLASH (v1.2.11). Quality filtering was performed in fastp (v0.23.1), chimeric sequences were removed using the SILVA 138.1 database and the VSEARCH package (v2.16.0), and final denoising was performed in QIIME2 with the DADA2 algorithm, obtaining amplicon sequence variants (ASVs). The nucleotide sequences of the identified bacteria were deposited in the NCBI (National Center for Biotechnology Information) database and are available at the following link: https://www.ncbi.nlm.nih.gov/nuccore/?term=PZ433566-PZ435422[accn] (accessed on 19 July 2026).

### 4.5. Data Processing and Statistical Analyses

Statistical analyses were performed using R software (version 4.0.3). Differences in the relative abundance of bacterial taxa and alpha diversity among experimental groups were evaluated using analysis of variance (ANOVA) or the non-parametric Kruskal–Wallis test, depending on data distribution. For effects reaching statistical significance, a post hoc t-test analysis was performed. For multiple comparisons, *p*-values were adjusted using the Benjamini–Hochberg method to control the false discovery rate (FDR). Statistical significance was set at *p* < 0.05. The obtained results were subjected to statistical analysis using the following tools and software: Statistica 13.3 [85] for *p* ≤ 0.05, TBtools-II v2.310 [86], SRplot (https://www.bioinformatics.com.cn) [87], and Chiplot (https://chiplot.online/) [88]. TBtools-II was used to generate a heatmap of the relative abundance of bacteria at the genus level. Bacterial metabolic function analyses were performed using FAPROTAX [89], and the results were presented as a heatmap in Chiplot. This tool was also used to visualize the number of bacterial ASVs, alpha- and beta-diversity indices, and plant parameters such as aboveground and root biomass and the leaf greenness index of *A. cruentus*. The number of bacterial ASVs at the genus level was presented on a dumbbell plot, alpha–diversity (Chao1, Shannon, Simpson, Pielou) as a heatmap, beta–diversity (Bray–Curtis, Jaccard) as a rose plot, and plant parameters using box plots.

## 5. Conclusions

Perfluorotetradecanoic acid significantly altered the soil bacterial microbiome through the selection of tolerant taxa and the elimination of sensitive ones. This compound promoted the growth of members of the phylum *Pseudomonadota* (including bacteria of the genera *Sphingomonas*, *Bradyrhizobium*, *Pseudolabrys*, and *Lysobacter*), accompanied by a decline in the relative abundance of members of *Actinomycetota*, *Chloroflexota*, and *Gemmatimonadota*, leading to reduced diversity and alterations in the structure of bacterial communities. At the same time, PFTeDA significantly modified both alpha and beta diversity, indicating changes in taxonomic richness and evenness, as well as greater dissimilarity among bacterial communities. Functionally, these changes were associated with shifts in the predicted metabolic potential of microbial communities, suggesting a possible reduction in functions related to humification and carbon cycling, while maintaining predicted functional capacities involved in nitrogen transformations and the degradation of organic compounds. The application of the biostimulants Shigeki and Aminoprim partially mitigated the effects of contamination by stabilizing the structure of the bacterial microbiome and supporting the recovery of selected taxa. Shigeki promoted the development of bacteria belonging to the phyla *Bacillota* and *Myxococcota*, whereas Aminoprim stimulated bacteria from the phyla *Pseudomonadota* and *Bacteroidota*. In the case of *Amaranthus cruentus*, perfluorotetradecanoic acid (PFTeDA) limited aboveground biomass accumulation without causing significant changes in chlorophyll content. The biostimulants enhanced biomass production and improved the functioning of the root system and photosynthetic apparatus, thereby increasing plant tolerance to chemical stress and supporting growth under soil contamination conditions. The results obtained advance our understanding of the mechanisms by which long-chain PFASs affect the soil environment and underscore the necessity of incorporating microbiological analyses into comprehensive environmental risk assessments. Furthermore, they identify biostimulation as a promising approach for supporting the remediation of PFTeDA-contaminated soils and the restoration of their ecosystem functions. A key direction for future research is the validation of these findings under field conditions and in soils with diverse physicochemical properties. Additional studies are required to assess the long-term effects of PFTeDA on soil microbial communities, the dynamics of biochemical processes, and the overall functioning of soil ecosystems. Moreover, a comprehensive evaluation of biostimulant efficacy is needed, including the optimization of application rates and timing, as well as a detailed investigation of their mechanisms of action. Such studies will be critical for determining the potential role of biostimulants as components of remediation strategies for PFAS-contaminated soils.

## Figures and Tables

**Table 1:** Characteristics of perfluorotetradecanoic acid [84].

Perfluorotetradecanoic Acid (PFTeDA)	
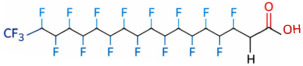 Structural formula	
**Parameter**	**Value/Formula**
Chemical formula	CF_3_(CF_2_)_12_COOH
Physical state	Solid
Molar mass	714.11 g mol^−1^
Density	0.89–1.8 g cm^3^
Melting point	130–135 °C
Boiling point	approx. 270 °C
Flash point	approx. 120–192 °C
Acute toxicity (oral)	500.1 mg kg^−1^
Acute toxicity (inhalation—vapor)	11.1 mg dm^−3^

**Table 2:** PCR primers used for bacterial DNA amplification.

Gene	Amplified Region	Primer	
		Forward	Reverse
16S rRNA	V4	GTGCCAGCMGCCGCGGTAA	GGACTACHVGGGTWTCTAAT
	V3–V4	CCTAYGGGRBGCASCAG	GGACTACNNGGGTATCTAAT
	V4–V5	GTGCCAGCMGCCGCGGTAA	CCGTCAATTCCTTTGAGTTT
	V5–V7	AACMGGATTAGATACCCKG	ACGTCATCCCCACCTTCC

## Data Availability

The original contributions presented in this study are included in the article. Further inquiries can be directed to the corresponding author.

## Abbreviations

The following abbreviations are used in this manuscript:

PFAS	Perfluoroalkyl and polyfluoroalkyl substances
PFTeDA	Perfluorotetradecanoic acid
C	Control soil
P	Soil treated with perfluorotetradecanoic acid
C_S	Soil with the addition of the biostimulant Shigeki
C_A	Soil with the addition of the biostimulant Aminoprim
P_S	Soil treated with perfluorotetradecanoic acid and with the addition of the biostimulant Shigeki
P_A	Soil treated with perfluorotetradecanoic acid and with the addition of the biostimulant Aminoprim
ASV	Amplicon Sequence Variant
SPAD	The leaf greenness index
